# A Hybrid Cryptosystem Incorporating a New Algorithm for Improved Entropy

**DOI:** 10.3390/e26020154

**Published:** 2024-02-10

**Authors:** Víctor Manuel Silva-García, Rolando Flores-Carapia, Manuel Alejandro Cardona-López

**Affiliations:** 1 Centro de Innovación y Desarrollo Tecnológico en Cómputo, Instituto Politécnico Nacional, Ciudad de México 07738, Mexico; vsilvag@ipn.mx (V.M.S.-G.); rfloresca@ipn.mx (R.F.-C.); 2 Centro de Investigación en Computación, Instituto Politécnico Nacional, Ciudad de México 07738, Mexico

**Keywords:** blockchain, Diffie–Hellman protocol, dynamic permutation, dynamic S-box, ElGamal system, entropy, Pi number

## Abstract

Today, safeguarding sensitive content through encryption is crucial. This work presents a hybrid cryptosystem for images that employs both asymmetric and symmetric encryption. The asymmetric component involves applying the Diffie–Hellman protocol and the ElGamal cryptosystem to securely transmit two constants. These constants are necessary for the symmetrical aspect to generate dynamic permutations, substitution boxes, and round keys. Following an encryption process with fourteen rounds, the encrypted images are processed by an algorithm proposed to enhance entropy, a critical metric for assessing encryption quality. It increases the frequencies of the basic colors to achieve a histogram closely resembling a uniform distribution, but it increases the image size by approximately 8%. This improves the entropy values achieved by the hybrid cryptosystem, bringing them remarkably close to the ideal value of 8.0. In specific instances, the entropy values were elevated from 7.99926 to 8.0. The proposed method exhibits resilience against various attacks, including differential, linear, brute force, and algebraic attacks, as evaluated through the entropy, correlation, goodness of fit, Discrete Fourier Transform (DFT), Number of Pixels Change Rate (NPCR), Unified Average Changing Intensity (UACI), Avalanche Criteria (AC), contrast, energy, and homogeneity. Further, encrypted images are subjected to noise attacks ranging from 20% to 50% noise, including additive, multiplicative, occlusion noise, as well as the newly introduced $\chi^{2}$ noise. The noise damage is quantified using the proposed Similarity Parameter (SP), and a 3 × 3 median filter is employed to enhance the visual quality.

## 1. Introduction

In this research paper, a hybrid cryptosystem is proposed to encrypt color images and facilitate key distribution. It includes an additional algorithm to enhance entropy, achieving values remarkably close to the ideal 8.0 and, in some instances, values of precisely 8.0. The described proposal involves augmenting the resulting encrypted image’s frequencies to achieve a histogram closely resembling a uniform distribution. This modification increases the image size by 8%. A detailed explanation of the algorithm is provided in Section 3.1. The cryptosystem features a symmetrical design of fourteen rounds, with the application of a distinct 8×8 S-box in each one. The process starts with a permutation and setting the key size to match the image dimensions. Additionally, the cryptosystem is dynamic; two constants multiplied by π generate permutations, S-boxes, and round keys that change in each encryption cycle. We named the proposal HAICDHBC, which stands for Hybrid Information Encryption Algorithm using the Diffie–Hellman Protocol and Blockchain. It enables information encryption, as any message can be represented by an image and subsequently encrypted. The distribution of constants is facilitated by the Diffie–Hellman protocol and the ElGamal asymmetric cryptosystem [1], establishing a robust key space.

Various hybrid cryptosystems have been developed to encrypt information [2,3,4,5,6]. However, none of them demonstrate an encryption quality that achieves entropy values remarkably close to eight. In contrast, our proposed system attains a value of eight for some images. Additionally, this study addresses the resistance of encrypted images against noise attacks. To assess the security of HAICDHBC, three types of attacks are studied in this paper: attacks applicable to the ElGamal cryptosystem, symmetric cryptosystems, and those designed to damage encrypted images. Attacks on the ElGamal asymmetric cryptosystem involve solving the discrete logarithm problem, which requires finding the value *a* in the equation $\alpha^{a}\;\mathrm{mod}\;p\equiv \beta$ given the values of β,α, and *p* [7,8]. Meanwhile, attacks on the symmetric cryptosystem include linear, differential, algebraic, and brute force methods [9,10,11].

Finally, the attacks on encrypted images involve the application of noise, including additive, multiplicative, and occlusion noise and noise with a $\chi^{2}$ distribution. Subsequently, HAICDHBC’s resistance to attacks is demonstrated. Additionally, images entirely in black and white are encrypted for evaluation, which is necessary as we use a symmetric cryptosystem. The randomness measurements of the encrypted images are reported in the results section.

The review of related works and their main differences to HAICDHBC commences with the entropy parameter. Although some cryptosystems report high entropies [12,13,14], the results achieved with HAICDHBC are superior. Similarly, the goodness-of-fit test values in other works on image encryption typically hover around 200 [15,16,17], whereas the ideal value is 0. This proposed method attains values ranging between zero and one. Another significant difference is that two types of noise are typically applied to encrypted images, including occlusion [18,19,20]. In contrast, the HAICDHBC proposal incorporates four types of noise. Additionally, in conventional papers, the loss of sharpness in decrypted images is not measured after noise application [21,22,23]. However, in this work, the Similarity Parameter (SP) is introduced to evaluate the loss of sharpness due to damage to encrypted information. Evaluating the quality of encrypted images involves critical assessments through randomness measurements. Two widely used parameters for this purpose are entropy and correlation.

Additionally, the consideration of real-world applications plays a crucial role in cryptosystem design. For example, Song et al. proposed an arbitrary-size encryption scheme to efficiently protect a batch of images with varying sizes [24]. Additionally, to enhance the encryption efficiency, Song et al. incorporated a parallel image encryption algorithm using intra-bitplane scrambling [25]. On the other hand, in certain countries such as Mexico, regulations prohibit data loss in images [26]. While our proposal increases the encrypted image size by 8% and increases the entropy, during the decryption stage, it is restored to its original dimensions without any loss of data. On the contrary, when employed in formats such as JPEG in lossy compression mode, suboptimal results are frequently observed, characterized by entropy values around 7.90 [27].

This paper is organized as follows: It commences with an introduction, which presents some related works and provides a brief overview of the state of the art. Section 2 introduces the theoretical elements used in this research. Section 3 outlines the construction of the new theoretical tools incorporated into the cipher algorithm. In Section 4, the encryption procedure and the test images are detailed. Section 5 presents the various noise types and how they are applied to the ciphered images, along with a high-level description of the median filter 3 × 3 and the SP parameter. The results are presented in Section 6, and an analysis and discussion are provided in Section 7. Finally, Section 8 contains the conclusions and outlines future work.

## 2. Mathematical Background of HAICDHBC

### 2.1. Pi Number

As mentioned earlier, an *S*-box of size 8×8 and dynamic permutations are employed in this work, both of which are random. The bits to the right of the decimal point of π exhibit important properties, such as the random appearance of zeros or ones. Table 1 provides evidence that $P\left(x_{i}=0\right)=P\left(x_{i}=1\right)=0.5$ for every bit position i≥0.

The results in Table 1 demonstrate a trend toward 50% as larger values of *n* are considered. The percentage of zeros may be greater than or less than 50%. Therefore, there is no preference for zeros or ones. In other words, the percentages of zeros and ones consistently fluctuate around the 50% mark. Additionally, another crucial property of the number π is that it is a transcendental number [28].

### 2.2. Blockchain and Hash Functions

In this research, the SHA-512 algorithm is used in the blockchain technique. This procedure is used to send two constants of approximately $2^{512}$ bits. Additionally, the proposed cryptosystem HAICDHBC uses a seed generated by the Diffie–Hellman protocol and the ElGamal cryptosystem [29]. Applying the SHA-512 algorithm to plaintext results in a 512-bit string defines a function that is not one-to-one. The latter property makes it nearly impossible to retrieve plaintext given the 512-bit string [30].

The solution to this problem is referred to as a preimage [31]. In this context, the probability that another distinct plaintext produces the same 512-bit output string is at most 50%. This scenario is termed a collision. The percentage of attacks on the SHA-512 algorithm is calculated over a set of $2^{256}$ plaintexts [31]. This property is utilized in the process of sending two constants, given that the 512-bit strings are public.

### 2.3. ElGamal Cryptosystem and the Diffie–Hellman Protocol

As previously mentioned, the HAICDHBC system incorporates the Diffie–Hellman protocol based on the ElGamal cryptosystem [29]. The following provides a detailed explanation of these methods.

The process commences with the ElGamal asymmetric cryptosystem, which is grounded in the set of residues denoted as $Z_{p}^{*}$ = {1,⋯,p−1}. In this study, the construction of *p* is proposed as $p=2k\times q_{1}q_{2}+1$, where $q_{1},q_{2}$ are two primes of approximately $2^{512}$ each, and c=1,2,⋯ [32]. This approach is chosen because it facilitates the retrieval of the generating element α by identifying the prime factors of p−1 and ensures the simplicity of the process [32]. Moreover, the even integer 2k does not exceed four decimal digits when the prime *p* is on the order of $2^{1024}$. Additionally, the verification of high primality for a positive integer ending in 1, 3, 7, or 9 is achieved through the Miller–Rabin algorithm [33].

The expression 0<α<p−1 is utilized to compute the generator element with the objective of satisfying Equation (1), where the *q*s are the prime factors of (p−1).

(1) $$\alpha^{(p-1)/q}\neq 1\;\mathrm{mod}\;p$$

Once the prime *p* and the generator element α are determined, the Diffie–Hellman protocol can be implemented. Both the sender *A* and the receiver *B* randomly generate positive integers $a_{A},a_{B}$ such that $1<a_{A},a_{B}<p-1$. Subsequently, the sender performs the following calculation: $\beta_{A}=\alpha^{a_{A}}$ mod. *p*. Similarly, the receiver carries out the operation $\beta_{B}=\alpha^{a_{B}}$ mod. *p*. The sender then sends $\beta_{A}$ to the receiver, and the receiver sends $\beta_{B}$ to the sender. Subsequently, the sender computes $\beta =\beta_{B}^{a_{A}}$ mod. *p*. It is important to note that the receiver can also obtain $\beta =\beta_{A}^{a_{B}}$ mod. *p*. In this way, the value β serves as the seed for sending two constants.

The authors think it pertinent to provide an illustrative example with values that are not suitable for real-world implementation but serve to elucidate the procedure. For example, assume the two primes are $q_{1}=113$ and $q_{2}=127$. Thus, $p=2\left(1\right)\times \left(q_{1}q_{2}\right)+1$ = 28,703. Hence, the prime factors, *q*, of p−1 are 2, 113, and 127. With these considerations in mind and utilizing the generator α = 14,807, the computations for $\alpha^{p-1/q}$ all result in values different from 1. Additionally, it is worth noting that the private keys of the sender and receiver are $a_{A}=623$ and $a_{B}=421$. Consequently, $\beta_{A}$ = 14,009, $\beta_{B}$ = 23,442, and the seed is β=14,438.

### 2.4. Entropy

As this paper’s title suggests, the encrypted images exhibit a high entropy, a parameter used to measure the their quality. Therefore, a brief overview of this parameter is presented. This method of measuring randomness, attributed to Shannon [34], is calculated according to Equation (2). Indeed, many works in the field of information encryption employ this measure [35].

(2) $$E\left(x\right)=-\sum_{x\varepsilon X}P_{r}\left(x\right)log_{2}P_{r}\left(x\right)$$

An encrypted image is deemed to have a good encryption quality if its entropy is close to 8, considering that each basic color has 256 intensity levels. To verify this assertion, in this work, ten additional measurements are used to confirm this characteristic.

### 2.5. Correlation Coefficient

The second parameter to discuss is the correlation coefficient. The correlation analysis of an encrypted image is conducted as follows: *m* pixels are randomly selected from the encrypted image. It is important to note that each pixel has three basic colors: red, green, and blue. Subsequently, the correlation is computed over the previously selected adjacent pixels. These pixels are considered in the horizontal, vertical, and diagonal directions [36]. With this information, Equation (3) is employed to calculate the correlation. It is worth mentioning that the subscript *c* indicates the color, and the subscript *d* signifies the direction. In this context, for a given direction, the variable $x_{i,c}$ in Equation (3) represents an intensity value such that $0\leq x_{i,c}\leq 255$.

The expressions of the variables $\bar{x}$ and $\bar{z}$ are defined in Equations (4) and (5). Also, it is important to note that an image is considered well encrypted if the correlation is a number close to zero [36].

(3) $$r_{d}=\frac{\frac{1}{n}\left(\sum_{i=1}^{n}\left(x_{i,c}-{\bar{x}}_{c}\right)\left(z_{i,c}-{\bar{z}}_{c}\right)\right)}{\sqrt{\frac{1}{n^{2}}\left(\sum_{i=1}^{n}{\left(x_{i,c}-{\bar{x}}_{c}\right)}^{2}\right)\left(\sum_{i=1}^{n}{\left(z_{i,c}-{\bar{z}}_{c}\right)}^{2}\right)}}$$

(4) $${\bar{x}}_{c}=\frac{1}{n}\sum_{i=1}^{n}x_{i,c}$$

(5) $${\bar{z}}_{c}=\frac{1}{n}\sum_{i=1}^{n}z_{i,c}$$

### 2.6. Discrete Fourier Transform

The Discrete Fourier Transform (DFT) is a statistical hypothesis test. It is commonly employed to quantify the degree of randomness in encrypted information [37]. This tool specifically scrutinizes the presence of repetitive bit strings. Additionally, it is worth highlighting that this parameter is incorporated into the NIST 800-22 standard [38]. In the computation of this parameter, the variables defined in Equations (6)–(8) are utilized, where *m* represents the length of the analyzed string, $M_{0}$ in Equation (6) is a constant value, and *l* in Equation (7) is a boundary.

(6) $$M_{0}=\frac{(0.95)\times m}{0.05}$$

(7) $$l=\sqrt{\mathrm{Ln}\frac{1}{0.05}\left(m\right)}$$

In this context, to compute the values of the functions $f_{j}$ appearing in Equation (8), it is important to consider that $y_{k}$ takes values of −1 and 1, while the complex unit is denoted as $i=\sqrt{-1}$. Additionally, $j=1,2,\ldots ,\frac{m}{2}-1$, taking into account that *m* is even, as it is the number of pixels expressed in bytes. Regarding the variable $N_{1}$ in Equation (9), its initial value is zero, i.e., $N_{1}=0$. Subsequently, $\|f_{j}\|$ is computed for each *j*, and the result is compared with *l*. If it is less than *l*, 1 is added to $N_{1}$; otherwise, the value of $N_{1}$ remains unchanged.

(8) $$f_{j}=\sum_{k=1}^{m}y_{k}e^{\frac{2\pi (i)(k-1)j}{n}}$$

After computing $\|f_{j}\|$ for all *j* and obtaining the final value of $N_{1}$, the variable *d* can be calculated using Equation (9). Like in all statistical hypothesis tests, there is a rejection region and an acceptance region. In this context, the variable *p*-value, as expressed in Equation (10), is taken as the decision parameter. If the *p*-value is less than 0.01, the hypothesis of randomness is rejected; otherwise, it is accepted. For this research, a significance level of 0.01 is considered [39].

(9) $$d=\frac{N_{1}-N_{0}}{\sqrt{\frac{m(0.95)(0.05)}{4}}}$$

(10) $$p-value=\mathrm{erfc}\frac{|d|}{\sqrt{2}}$$

Additionally, the erfc function is evaluated as Equation (11).

(11) $$\mathrm{erfc}\frac{|d|}{\sqrt{2}}=2\left(1-\Phi \left(|d|\right)\right)$$

### 2.7. Goodness-of-Fit Test

Similar to the previous parameter, the procedure to measure the goodness of fit is a statistical hypothesis test. It assesses if the information conforms to a uniform distribution for each of the basic colors.

In this context, the null hypothesis posits that the string of bits is random, while the alternative hypothesis asserts the opposite. It is essential to note that in every hypothesis test, a statistic is formulated, and a rejection region is defined based on the chosen level of significance [40].

The goodness of fit is defined in Equation (12). Furthermore, it follows a $\chi^{2}$ distribution with n−1 degrees of freedom.

(12) $$\chi^{2}=\sum_{i=1}^{n}\frac{{\left(o_{i}-\exp\right)}^{2}}{\exp}$$

Additionally, based on the central limit theorem, the variable $\chi^{2}$ converges to a normal distribution with a mean of μ=255 and a variance of σ=22.5 [41]. For a significance level of α=0.01, the decision rule is as follows: if $\chi^{2}\leq 308$, the null hypothesis is accepted; otherwise, it is rejected. On the other hand, note that this type of instrument is not included in the NIST 800-22 standard when testing the randomness of a bit string.

### 2.8. NPCR, UACI, and AC Parameters

The resistance of HAICDHBC against a differential attack is measured using the Number of Pixels Change Rate (NPCR), Unified Average Changing Intensity (UACI), and Avalanche Criteria (AC) parameters. Each of them is briefly described below.

The NPCR parameter is defined according to Equation (13), where the subscript *c* indicates the analyzed color, and *W* and *H* are the width and height of the encrypted image, respectively. Additionally, the function D(i,j) evaluates the differences between two encrypted images denoted as 1 and 2, both with the same width and height. Given a position (i,j), the pixels of images 1 and 2 are compared at this position. If both pixels are equal, D(i,j)=0; otherwise, D(i,j)=1. When this parameter approaches a value of 99.6%, the encryption is considered to be resistant to a differential attack [42].

(13) $${\mathrm{NPCR}}_{c}=\frac{\Sigma_{i,j}D{\left(i,j\right)}_{c}}{W\times H}\times 100\%$$

The UACI parameter also assesses the difference between two images. In this case, it considers the variations in intensities of each pixel, which are integers ranging from 0 to 255. The UACI is determined using Equation (14). It is important to note that the subscripts 1,c and 2,c indicate the image number and the specific basic color being utilized. Additionally, the variables *W* and *H* represent the width and height of the analyzed images. Furthermore, the value considered desirable for this parameter to mitigate the impact of a differential attack is 33.4% [43].

(14) $${\mathrm{UACI}}_{c}=\frac{1}{W\times H}\sum_{i,j}\left[\frac{|I_{1,c}\left(i,j\right)-I_{2,c}\left(i,j\right)|}{255}\right]\times 100\%$$

The third parameter, AC, is determined according to Equation (15). In this expression, *T* represents the size of all image pixels in bits. Additionally, the subscript *c* designates the color. Thus, this parameter assesses the differences, bit by bit, between images 1 and 2. The function d(i,j) in Equation (16) takes the value 0 when the bits at position (i,j) in both images are the same and 1 otherwise. A desirable value for AC is considered to be 50%.

(15) $${\mathrm{AC}}_{c}=\frac{\Sigma_{i,j}d{\left(i,j\right)}_{c}}{T}\times 100\%$$

(16) d(i,j)c=01

### 2.9. Homogeneity, Contrast, and Energy

In this part, a high-level description of the homogeneity, contrast, and energy parameters is given. Homogeneity is calculated using Equation (17), where the function g(i,j) indicates the value it takes at the point (i,j). On the other hand, an encrypted image is considered to be of high quality if the homogeneity is low [44].

(17) $$Homogeneity=\sum_{i,j}\frac{g(i,j)}{1+|i-j|}$$

The contrast parameter is assessed using Equation (18). In this context, contrast quantifies the variations between adjacent points (i,j). Similarly to before, f(i,j) represents the value of *f* at the point (i,j). It is worth noting that an image is considered to be well encrypted when the contrast values are large [45].

(18) $$Contrast=\sum_{i,j}|i-j{|}^{2}g\left(i,j\right)$$

To conclude this section, we will discuss the energy parameter, measured using Equation (19). This parameter assesses the level of information disorder in an encrypted image. An image is considered well encrypted when the energy is close to zero [46].

(19) $$Energy=\sum_{i,j}g{\left(i,j\right)}^{2}$$

### 2.10. The Median Filter

Following noise damage to encrypted images, a 3 × 3 filter tool is employed after decryption. Subsequently, the sharpness enhancement in the impaired images is quantified using the SP parameter.

**Figure 1:**
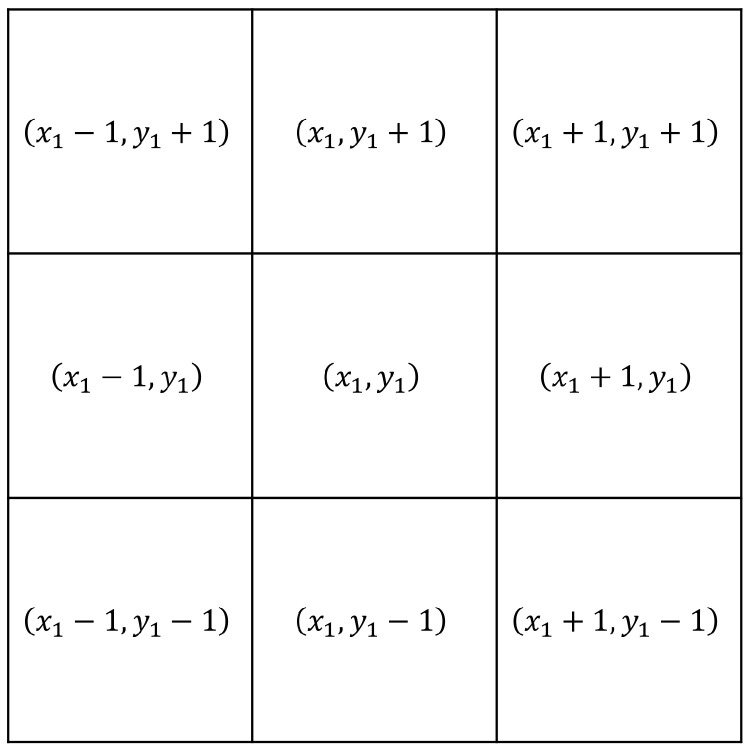
A 3 × 3 median filter.

This filter application is a non-linear procedure [47]. It involves constructing a nine-point mask around a pixel $\left(x_{1},y_{1}\right)$ in the decrypted image affected by noise, as illustrated in Figure 1. The pixels in the mask are arranged based on intensity, and the median value is selected. This median value, denoted as $M_{c,(x_{1},y_{1})}$, with *c* indicating the basic color, must be greater than or equal to the first $\lceil \frac{9}{2}\rceil -1$ pixels and less than the remaining ones.

After obtaining the median value, it is substituted for each pixel in the nine-point mask.

## 3. Development of New Elements

In this section, we will use the proposed algorithm on an encrypted image to enhance the entropy. Additionally, the algorithm for generating permutations and the Similarity Parameter (SP) will be introduced. To commence, we will outline the algorithm designed to augment entropy.

### 3.1. Algorithm to Enhance Entropy

The entropy-enhancement algorithm begins by denoting an encrypted image as *A*. Its dimensions are 512 × 512 pixels and it has a discrete area ∣A∣ equal to 262,144 pixels. We will detail the algorithm used to encrypt images in Section 4. With this in mind, it is important to highlight that it is possible to obtain three color histograms from the encrypted image. Each one comprises 256 intensities *i* in the range of 0≤i≤255. The frequency of each intensity is denoted as follows: $f_{r,i}$, $f_{g,i}$, and $f_{b,i}$, where *r*, *g*, and *b* represent the basic colors *c*, and *i* is the intensity. Additionally, it holds true for each color that $\sum_{i=0}^{255}f_{r,i}=\sum_{i=0}^{255}f_{g,i}=\sum_{i=0}^{255}f_{b,i}$ = 262,144. As part of the proposed method, the size of the encrypted image is increased by approximately 8% to improve the encryption quality. The advantages of this enhancement will be presented in Section 6. To achieve this, rows of 512 pixels are added to the encrypted image *A* after row 511. Image enlargement $A^{\prime }$ finishes when the number of pixels is greater than or equal to ∣A∣× 1.08. Let us denote the increased discrete area with *n* new rows as $|A^{\prime }|$. The value of each frequency in the enlarged image is around the value *h* defined in Equation (20).

(20) $$h=\frac{|A^{\prime }|}{256}$$

Subsequently, the difference $d_{c,i}$ between the values *h* and the frequency $f_{c,i}$ of the encrypted image *A* is defined. Specifically, $d_{r,i}=h-f_{r,i}$, $d_{g,i}=h-f_{g,i}$, and $d_{b,i}=h-f_{b,i}$, where 0≤i≤255. The variable *d* can be greater than, equal to, or less than zero. Given these variables, the steps of the algorithm developed in this paper to obtain the increased part of *A* are as follows, and this process is replicated for each basic color.

1. First iteration. The frequency $f_{c,0}$ has an associated difference $d_{c_{0}}$. When this difference is greater than zero, one is added to the frequency $f_{c,0}$; otherwise, the frequency remains unchanged. This strategy continues for $f_{c,2}$ until $f_{c,255}$.
2. Consecutive iterations. The process restarts with the first frequency, which might have been modified in the previous iteration. Therefore, the difference *d* is recalculated, and $f_{c,0}$ is modified according to the result. The process is executed in the same manner as before for all frequencies, while updating $d_{c,i}$. This iterative process continues until the sum of the added pixels equals *n* × 512, which is equivalent to the number of pixels in the added rows.

To conclude the algorithm, three permutations are applied, one for each basic color of the pixels that were increased. This is executed to ensure that the enlarged part is an image color. Once this is completed, another permutation *P* is applied to the entire enlarged image $A^{\prime }$.

### 3.2. Algorithm for Constructing Permutations

As indicated in the previous section, the method proposed in this work involves permutations. Furthermore, it is asserted that any non-negative integer can be expressed on a factorial basis. In this context, the set of non-negative integers is defined as $Z_{m}=\left\{n\in N|0\leq n\leq m!-1\right\}$ for a given m≥2.

Hence, any element $n_{0}$ of the set $Z_{m}$ can be expressed in the factorial base (m−1)!,(m−2)!⋯1!,0! This is illustrated in Equation (21):

(21) $$n_{0}=D_{0}\left(m-1\right)!+D_{1}\left(m-2\right)!+\ldots D_{m-2}\left(1\right)!+D_{m-1}\left(0\right)!$$

Also, according to Euclid’s division algorithm, the $D_{i}$ coefficients in Equation (21) are unique [48]. It will be shown later that $D_{m-1}=0$. Furthermore, the coefficients of Equation (21) satisfy the inequality in Equation (22).

(22) $$0\leq D_{i}<\left(m-i\right)\mathrm{with}0\leq i\leq \left(m-2\right)$$

Taking into account Equations (21) and (22), an algorithm is constructed to obtain permutations on arrays of *m* positions [49]. Also, note that the (m−i)! values appear as factors in Equation (21) because, in a 512 × 512 image, there are 262,144 placements, making it impractical to write at 250,000!, at least for now.

To conclude this section, it is noted that the algorithm to construct the permutations defines a one-to-one function [49].

### 3.3. Similarity Parameter

Encrypted images are susceptible to noise, and thus they may appear distorted when decrypted. Therefore, it is advantageous to devise a parameter that quantifies the loss in sharpness [50]. For this, in this paper, the parameter ${\mathrm{SP}}_{c}$ is introduced to assess the degradation of decrypted images. Specifically, Equation (23) defines ${\mathrm{SP}}_{c}$, with the subscript indicating the basic color under analysis.

(23) $${\mathrm{SP}}_{c}=\left|\left[100\%-{\mathrm{UACI}}_{c}\left(2.994011\right)\right]\right|$$

It is based on the UACI parameter, previously defined in Equation (14), which assesses the distinction between two images. In this context, two extreme cases are presented below to describe the SP performance.

In the first case, a plain image is compared with an encrypted one to simulate the total noise damage. If a figure is well encrypted, UACI ≅33.4% [43], and consequently, ${\mathrm{SP}}_{c}=\left|\left[100\%-33.4\%\left(2.994011\right)\right]\right|=0.036\%≅0\%$. This would indicate a total loss of sharpness, signifying complete information loss.

In the second case, if both images being compared are the same, it is implied that UACI = 0%. Consequently, ${\mathrm{SP}}_{c}=\left|\left[100\%-0\%\left(2.994011\right)\right]\right|=100\%$. However, this scenario signifies that both images are equal, and there is no information loss. In conclusion, ${\mathrm{SP}}_{c}$ measures the sharpness from 0% to 100%.

To summarize this section, this tool will be employed in the present work to assess the improvement in sharpness after applying the 3 × 3 filter to the damaged images.

## 4. Encryption Procedure

The hybrid encryption cryptosystem comprises two cryptosystems: one asymmetric cryptosystem and another symmetric cryptosystem. We will now present a description of the asymmetric cryptosystem.

### 4.1. Asymmetric Cryptosystem

Two integer constants, denoted as $C_{1}$ and $C_{2}$, are initially proposed with the condition $0<C_{1},C_{2}\leq 2^{512}$. Subsequently, the asymmetric ElGamal cryptosystem and the SHA-512 algorithm are employed to transmit these constants [51]. The process begins with the ElGamal cryptosystem, where the sender possesses knowledge of the receiver’s public key $\beta_{B}$ and the receiver is aware of the sender’s public key, $\beta_{A}$.

It is essential to consider that $\beta_{B}=\alpha^{a_{B}}\mathrm{mod}.p$ and $\beta_{A}=\alpha^{a_{A}}\mathrm{mod}.p$, where $a_{A}$ and $a_{B}$ are private while α and *p* are public parameters. Both the sender and the receiver possess the knowledge of $\beta ={\left(\beta_{B}\right)}^{a_{A}}\mathrm{mod}.p$ and $\beta ={\left(\beta_{A}\right)}^{a_{B}}\mathrm{mod}.p$.

With this information, the following steps are executed:

1. The sender generates two constants, denoted as $C_{1}$ and $C_{2}$, each being a 512-bit string $0<C_{1},C_{2}\leq 2^{512}$. If the representation of the constants is shorter than 512 bits, the sender pads zeros to the left to ensure that the length remains at 512 bits.
2. $\beta_{i}$ is computed using the formula $\beta_{i}=\alpha^{i}\times \beta ,\mathrm{mod}.,p$ for i=1,2,⋯,128. It is important to note that the initial 64 $\beta_{i}$ values are designated for transmitting $C_{1}$, while the subsequent 64 $\beta_{i}$ values are intended for sending $C_{2}$.
3. The constants are transmitted via the following process: The 512-bit string corresponding to $C_{1}$ is segmented into one-byte blocks, resulting in 64 blocks. Each block is associated with an integer $b_{i}$ ranging from 0 to 255. If the *i*-th byte has a value of zero, the SHA-512 algorithm is applied once to $\beta_{i}$, SHA-512($\beta_{i}$). Conversely, if the value of $b_{i}$ falls within the range of $1\leq b_{i}\leq 255$, the SHA-512 algorithm is iteratively applied $b_{i}+1$ times to the string $\beta_{i}$, yielding a 512-bit string, which is public.
4. The receiver computes $\beta_{i}$ and sequentially applies the SHA-512 algorithm to each $\beta_{i}$, given that they possess knowledge of β. Consequently, the receiver can determine the values of $b_{i}$ and retrieve the constants $C_{1}$ and $C_{2}$.

### 4.2. Symmetric Cryptosystem

The symmetric encryption procedure comprises two stages. Initially, the plain image undergoes encryption through fourteen rounds. Subsequently, the encrypted image is expanded following the algorithm detailed in Section III. Here, a high-level description outlines the processes in the symmetric cryptosystem during the initial stage. Additionally, the construction of the involved elements is illustrated as follows:

1. First Round. The process commences with an XOR operation between the original image pixels and the first round key. The resulting chain is then segmented into one-byte blocks. Subsequently, substitution is implemented following the procedure established by the Advanced Encryption Standard (AES). This process utilizes the first of the fourteen dynamic substitution boxes.
2. Rounds two to fourteen. The same process is replicated, involving the byte chain from the previous round and the corresponding round key. The resulting string is then processed through the appropriate box, following the protocol established in the previous step. During round fourteen, three operations are performed: the XOR operation using the fourteen round-key, passing the result through the fourteenth box. In the third step, an XOR operation is executed between the chain emerging from the boxes and the fifteen round-key. This final result is considered the initial stage of image encryption.

The generation of boxes, permutations, and rounds keys is detailed below.

- Substitution box. Each substitution box is a permutation of 256 values ranging from 00 to ff in a hexadecimal system. The sender constructs the fifteen boxes used in the encryption process through the following steps. First, compute $C_{1}\times \pi$ by considering the bits to the right of the decimal point. This bit string is then divided into one-byte blocks. Taking the first byte, representing an integer $c_{0}$, calculate $D_{0}$ = $c_{0}$ mod. 256. For $D_{i}$, where the i−th byte to the right of the decimal point is $c_{i}$, compute $D_{i}$ = $c_{i}$ mod. 256−i. Once $D_{i}$ values are available for 0≤i≤255, apply the procedure in Section 3.2, which results in the first substitution box. For the j−th box, where 2≤j≤14, shifts of (j−1)×256 bytes are made to the right of the decimal point, and then the same process is applied as before.
- Permutation. The permutation *P*, applied at the end of the process, is constructed in the following way. The sender computes the product $C_{2}\times \pi$, and the bits to the right of the decimal point are then divided into bytes. Here, the calculation of the constant $D_{0}$ involves pixels 0, 1, and 2. This string of three pixels has an associated integer of 24 bits denoted as $d_{0}$, and let l be the number of pixels in the enlarged image. Therefore, $D_{0}$ = $d_{0}$ mod. *l*. To obtain the other constants, shifts of one pixel to the right are made. For instance, in the case of $D_{1}$, pixels 1, 2, and 3 are considered. Then, for the *i*-th coefficient, pixels i, i+1, and i+2 are considered, resulting in the integer $d_{i}$. Hence, $D_{i}$ = $d_{i}$ mod. l−i, where 1≤i≤l−2.
- Round keys. Round keys are 512 × 512 byte-size pixels. The first round key is calculated as follows: from the product $C_{2}\times \pi$, the first (512 × 512) × 24 bits to the right of the decimal point are taken. The reason for multiplying by 24 is the color images, where pixel representation is 24 bits (three bytes). Note that in the case of a 256-grayscale image, it is only multiplied by eight. This string is then divided into bytes and subsequently passed through the first substitution box, similarly to the AES procedure [52]. The chain that results from this process is denoted as $k_{1}$. In general, to generate the *i*-th round key $k_{i}$, we proceed as follows: a shift of i−1 bits is made to the right of the decimal point from $C_{2}\times \pi$, with 1≤i≤15. Afterward, the corresponding substitution box is applied, following the same rule as before. Note that for the round key $k_{15}$, box fourteen is used.

In addition, the receiver can reproduce this procedure once it knows $C_{1},C_{2}$, and therefore they can decrypt the image. Constants are generated randomly for every image encryption; this implies that boxes, permutations, and round keys are dynamic. This is possible as the function f(C)=C×π is a one-to-one function. In other words, if $C_{1}\neq C_{2}$, it is implied that $f\left(C_{1}\right)\neq f\left(C_{2}\right)$. This ensures that the bit strings on the right side of the decimal point are different when the constants change.

### 4.3. Second Stage

Three permutations are applied only to the incremented image part, one for each basic color. However, these permutations are not executed in the decryption process because this is just noise without information. On the other hand, it is noted that the three permutations are constructed in the same way as *P*, but the number of pixels, in this case, is *l* = *n* × 512, where *n* represents the number of incremented rows. After applying the three permutations, the permutation *P* is executed across the entire image, encompassing |A|×1.08 pixels. This step concludes the encryption process.

We present the values utilized in this study—$q_{1}$, $q_{2}$, *k*, $p^{*}$, α—and the private keys $\beta_{A}$ and $\beta_{B}$ of both the sender and receiver in Table 2.

### 4.4. Images for Testing

**Figure 2:**
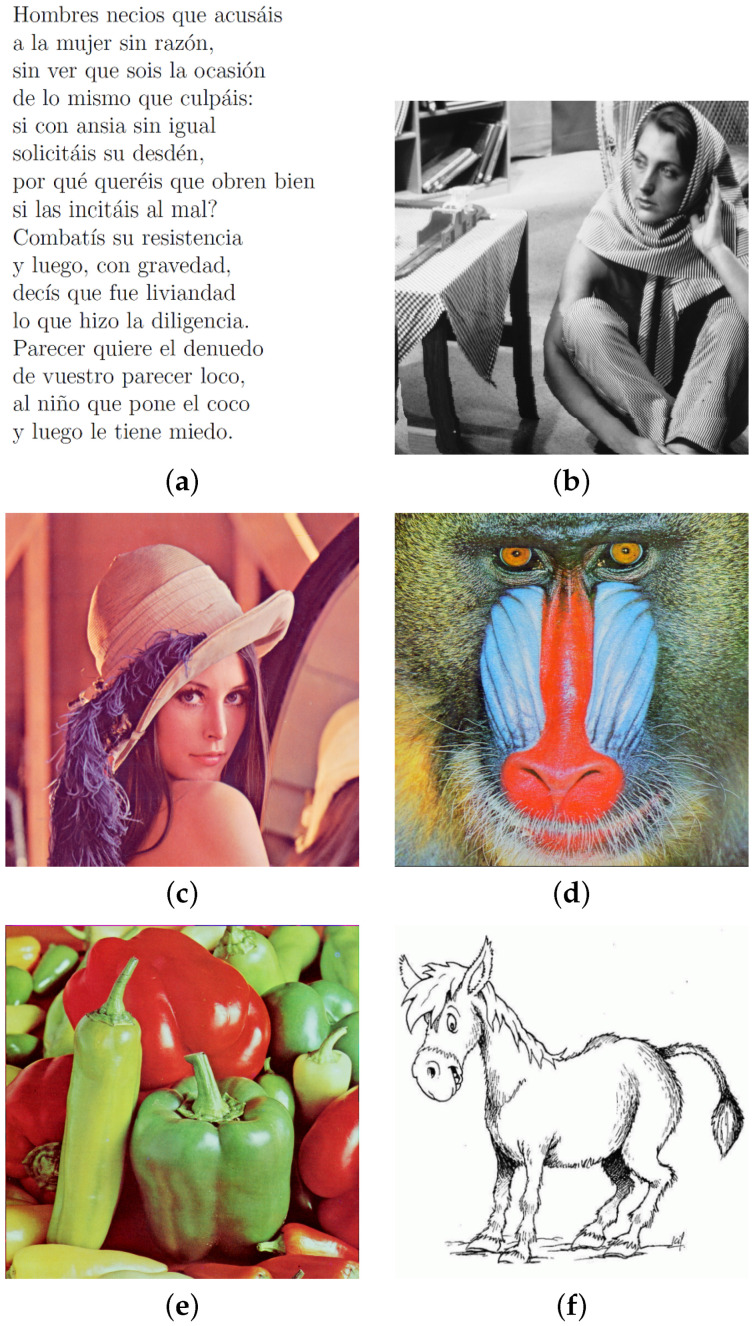
Images utilized for evaluating HAICDHBC. (**a**) Sor Juana. (**b**) Barbara. (**c**) Lena. (**d**) Baboon. (**e**) Peppers. (**f**) Donkey.

The performance of HAICDHBC was assessed with a range of images, presented in Figure 2. They consist of color and grayscale images and a message. The widely utilized Lena image is included, given its common use in image encryption studies [53]. Furthermore, two additional images, one entirely black and the other in white, will be considered in subsequent analyses.

Throughout this research, most plain images have dimensions of 512 × 512 pixels. It is worth mentioning that while this particular size was used in the proposed encryption algorithm, it can be used with images of varying dimensions. For instance, the Sor Juana image, which contains one of her poems, has a size of 423 × 544 pixels. Another noteworthy observation is that all images encrypted using HAICDHBC, whether in color or grayscale, yield a color figure as a result.

The performance of HAICDHBC is compared with AES-CBC for images affected by noise [54]. This comparison will be detailed in the following section.

## 5. Damaged Encrypted Images with Noise

In this study, the encrypted images are subjected to four types of noise attacks to test the HAICDHBC cryptosystem: additive, multiplicative, occlusion, and $\chi^{2}$ noise attacks. To elaborate on this, we will start by discussing additive and multiplicative noises.

### 5.1. Additive and Multiplicative Noises

A high-level description of both types of noises is provided. Initially, *n* random pixels are selected from the encrypted image, and each of these points is associated with a color level denoted as $g_{c}\left(x,y\right)$, where $0\leq g_{c}\left(x,y\right)\leq 255$. Here, the subscript *c* indicates the basic color.

To generate additive noise, a non-zero integer $\phi_{c}\left(x,y\right)$ is randomly chosen, depending on the point and basic color. Subsequently, the operations outlined in Equation (24) are executed, resulting in an integer $g_{c}^{\prime }\left(x,y\right)$ within the range of 0 to 255. To introduce damage to an image encrypted with additive noise, the value of $g_{c}\left(x,y\right)$ is replaced with $g_{c}^{\prime }\left(x,y\right)$.

(24) $$g_{c}^{\prime }\left(x,y\right)=\left[g_{c}\left(x,y\right)+\phi_{c}\left(x,y\right)\right]\;\mathrm{mod}\;256$$

In the case of multiplicative noise, much like additive noise, a non-zero integer $\phi_{c}\left(x,y\right)$ is randomly determined. Following this, Equation (25) is solved. To introduce damage using multiplicative noise, the color level $g_{c}\left(x,y\right)$ is then substituted with $g_{c}^{\prime }\left(x,y\right)$.

(25) $$g_{c}^{\prime }\left(x,y\right)=\left[g_{c}\left(x,y\right)\times \phi_{c}\left(x,y\right)\right]\;\mathrm{mod}\;256$$

### 5.2. Occlusion Noise

**Figure 3:**
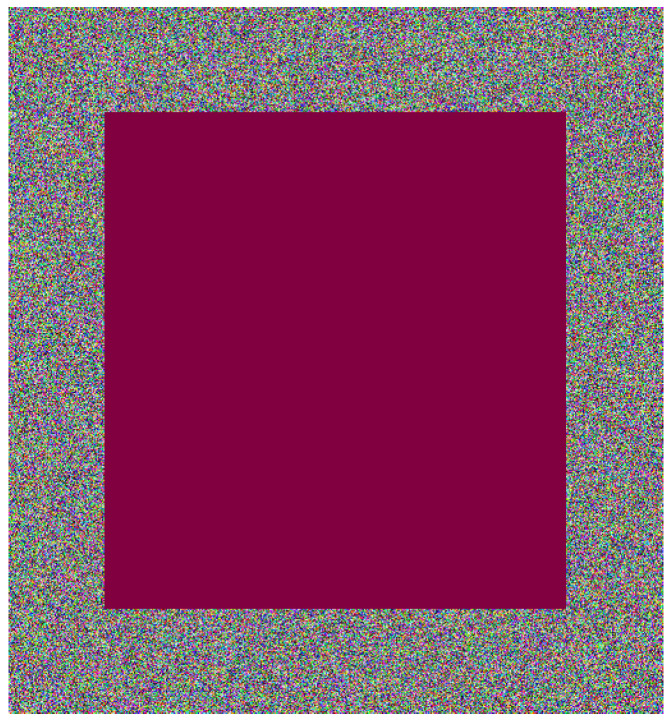
Encrypted Lena image affected by occlusion noise at 50%.

Occlusion noise involves damaging a confined area of an encrypted image. In this study, this noise is applied over a concentric parallelogram, as illustrated in Figure 3. Specifically, the color cherry is utilized, although another color could be used. The process involves substituting the pixel color at a point inside the parallelogram with cherry. Similar approaches have been employed in other research, although the shape may not necessarily be a parallelogram [55].

### 5.3. Chi-Square Noise

As previously mentioned, the proposed noise is referred to as $\chi^{2}$ noise based on the $\chi^{2}$ distribution. $\chi^{2}$ noise can be described by Equation (26). Additionally, the variable defined in Equation (12) follows a $\chi^{2}$ distribution with n−1 degrees of freedom [56]. However, this distribution approximates to a normal distribution N(μ,σ) because there are 256 color levels. Considering that n=256, it follows that mean μ=255 and standard deviation σ=22.58.

Subsequently, to apply $\chi^{2}$ noise, *m* pixels (x,y) from the encrypted image are randomly selected. Each of these pixels possesses a color level within the range of $0\leq g_{c}\left(x,y\right)\leq 255$, where the subscript *c* designates the basic color.

For each pixel and basic color, a randomly chosen value denoted as $z_{c}\left(x,y\right)$ is determined, following a standard normal distribution, expressed as $z_{c}\left(x,y\right)∼$ N(0, 1). The range of these values extends from −∞ to *∞*. However, in this study, we limit the values to the interval $-3<z_{c}\left(x,y\right)<3$ and use the following criterion: if $z_{c}<-3$, the value is assigned as −3; if $z_{c}>3$, it is set to 3.

With this information, the value of $g_{c}^{\prime }$ is calculated using Equation (26). The result is not necessarily an integer, and the symbols ⌊⌋ and ⌈⌉ are used to discretize it as $g_{dc}^{\prime }$. If the decimal part of $g_{c}^{\prime }\left(x,y\right)$ is less than or equal to 0.5, ⌊⌋ is applied, meaning that $g_{dc}^{\prime }$ takes only the integer part of $g_{c}^{\prime }\left(x,y\right)$. On the other hand, if the decimal part of $g_{c}^{\prime }\left(x,y\right)$ is greater than 0.5, then ⌈⌉ is used, indicating that the integer part of $g_{c}^{\prime }\left(x,y\right)$ plus one is taken by $g_{dc}^{\prime }$.

(26) $$g_{c}^{\prime }\left(x,y\right)=255+z_{c}\left(x,y\right)22.58$$

Now, to apply $\chi^{2}$ noise, the color level $g_{c}\left(x,y\right)$ is replaced by $g_{dc}^{\prime }\left(x,y\right)$ for all randomly chosen points.

To conclude this section, it is worth noting that when using this type of noise in the encrypted image, the majority of the randomly chosen pixels undergo a substitution with extreme values within the interval of 0–255, that is, values ranging from 0 to 64 or from 191 to 255.

## 6. Results

**Figure 4:**
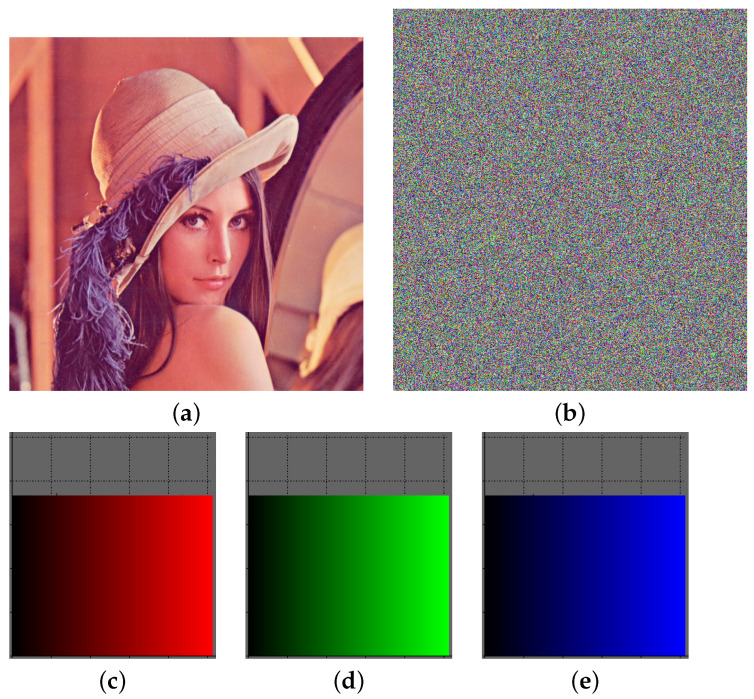
Lena encryption results. (**a**) Lena plain image. (**b**) Lena encrypted image. (**c**) Red histogram of (**b**). (**d**) Green histogram of (**b**). (**e**) Blue histogram of (**b**).

This section commences with the presentation of the Lena image in a flat state, as depicted in Figure 4a. In Figure 4b, the corresponding encrypted outcome is showcased. It is discernible that the encrypted figure is expanded in comparison to the original. Additionally, the histograms of the basic colors red and green appear almost horizontal, while that of the color blue is completely horizontal. The histograms are presented in Figure 4c–e.

In terms of evaluations, we will initially show the results of the encrypted images without noise. The evaluations to be presented include entropy, correlation, NPCR, UACI, AC, contrast, homogeneity, and energy. Following that, we will present evaluations utilizing statistical hypothesis tests such as the Discrete Fourier Transform and the goodness-of-fit test.

With this in mind, the subsequent subsection presents the results of entropy and correlation.

### 6.1. Entropy and Correlation

It should be noted that the assessed images correspond to those shown in Figure 2. The purpose of presenting these results is to gauge the randomness of the encrypted images. Table 3 and Table 4 display the evaluations of entropy and correlation, respectively.

### 6.2. Differential Attack

The NPCR, UACI, and AC values are presented in Table 5, Table 6 and Table 7, respectively.

### 6.3. Energy, Contrast, and Homogeneity

Continuing with the presentation of results, we now focus on the following parameters: energy, contrast, and homogeneity. These are displayed in Table 8, Table 9 and Table 10, respectively.

### 6.4. The Goodness-of-Fit Test and Discrete Fourier Transform

This section presents the results of the hypothesis tests, specifically the goodness of fit based on the $\chi^{2}$ value and the Discrete Fourier Transform (DFT). The evaluations of both are displayed in Table 11 and Table 12.

### 6.5. Black and White Images

As the hybrid cryptosystem, HAICDHBC, includes a symmetric algorithm, it is beneficial to assess the encryption of two images: one entirely black image and another entirely white image. It should be noted that the size of both images is 512 × 512 pixels. The encrypted figures were evaluated using entropy and correlation; the results of these measurements are presented in Table 13.

### 6.6. Attack on Encrypted Images with Noise

**Figure 5:**
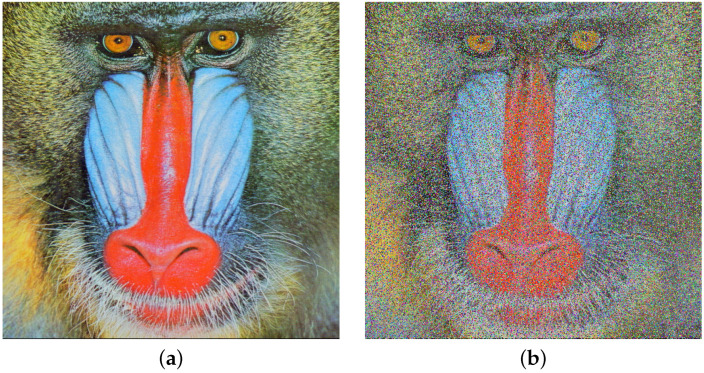
HAICDHBC resistance to $\chi^{2}$ noise. (**a**) Plain Baboon image. (**b**) Baboon decryption after the application of $\chi^{2}$ noise of 50 % in the encryption stage.

We will now present images subjected to noise after encryption. The procedure is illustrated with the Baboon image and implemented as follows. The original image is displayed in Figure 5a. Subsequently, the image undergoes encryption using HAICDHBC and is then subjected to $\chi^{2}$ noise with a magnitude of 50%. To finalize the process, the damaged image is decrypted, and the outcome is exhibited in Figure 5b.

**Figure 6:**
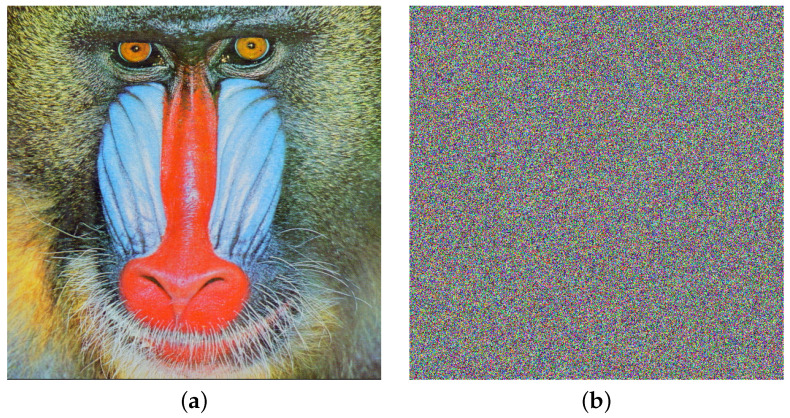
Advanced Encryption Standard (AES)-CBC resistance to additive noise. (**a**) Plain Baboon image. (**b**) Baboon image decryption after the application of additive noise of 50 % in the encryption stage.

Another experiment involving noise was conducted as follows: the Baboon image was encrypted, but this time using the standard AES-CBC. Subsequently, additive noise with a magnitude of 50% was applied to the encrypted figure. The damaged image was then decrypted using AES-CBC. The outcome of this procedure is illustrated in Figure 6. A discussion related to Figure 5 and Figure 6 is provided in the results analysis section.

**Figure 7:**
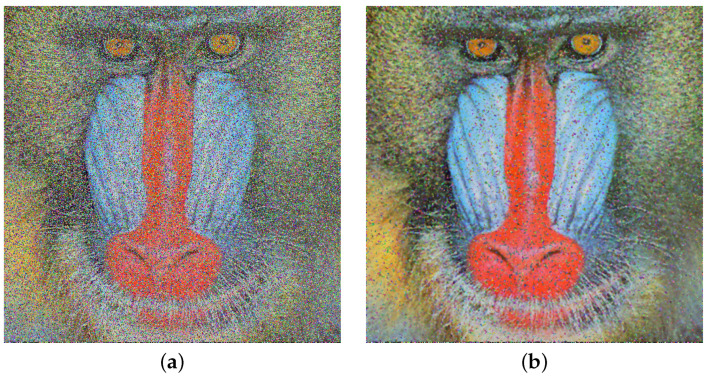
Baboon image enhancement visual quality. (**a**) Baboon image decryption with chi-square noise of 50%. (**b**) Decrypted Baboon image after a 3×3 filter application to (**a**).

Another crucial aspect studied in this section is the application of the median filter, which is employed to enhance the visual quality of images affected by noise. As described in Section 2.10, a 3 × 3 median filter was utilized. Figure 7a displays the Baboon image damaged by 50% $\chi^{2}$ noise, while Figure 7b exhibits the resulting image after applying the 3 × 3 median filter.

Following the presentation of the image results, evaluations using the SP parameter are now showcased. Table 14 displays the SP results for various sizes of $\chi^{2}$ noise applied to the images depicted in Figure 2.

To conclude this section, sharpness evaluations using the SP parameter are presented in Table 15. In this analysis, four types of noise were explored, with a fixed damage size of 50%. The images in Figure 2 were utilized in this process, with the application a 3 × 3 median filter after decryption.

## 7. Results Analysis and Discussion

In the security analysis of the proposed hybrid cryptosystem, the key-space in the asymmetric cryptosystem is first analyzed, followed by the symmetric key-space. In the asymmetric cryptosystem, the sender’s and receiver’s public keys, denoted as $\beta_{A}$ and $\beta_{B}$, respectively, satisfy the condition $1\leq \beta_{A},\beta_{B}\leq p-1$. Given that *p* is approximately $2^{1024}$, the key space for the asymmetric cryptosystem is on the order of $2^{1024}$ possible elements. For key construction in the symmetric cryptosystem, two random constants, $C_{1}$ and $C_{2}$, are chosen, such that $0\leq C_{1},C_{2}\leq 2^{512}$. Consequently, the number of keys in the symmetric cryptosystem is on the order of $2^{512}\times 2^{512}$, which is equal to $2^{1024}$. Therefore, the key space of the hybrid cryptosystem is estimated to be around $2^{1024}$.

The cryptosystem’s security is also analyzed for potential attacks on HAICDHBC due to its asymmetric and symmetric composition. First, attacks on the ElGamal asymmetric cryptosystem are considered. The objective of such attacks is to unveil the sender’s private key $a_{A}$ when the public key $\beta_{A}$ is known. Various generic algorithms, such as the Pohlig–Hellman attack, have been developed for this purpose, with a complexity of $O(\sqrt{p})$ [57]. Given that the prime used in this work is approximately $2^{1024}$, the complexity of such attacks would be on the order of $O(2^{512})$. Consequently, they are unfeasible, at least with existing technology.

For the symmetric system, the dynamic generation of the fourteen 8×8 substitution boxes in every encryption process ensures that they remain undisclosed. The latter avoids potential attacks, such as linear and algebraic attacks, at least as they are currently understood [9,58]. Regarding a differential attack, the results of NPCR, UACI, and AC in Table 5, Table 6 and Table 7 indicate that this type of attack ca be avoided.

Concerning noise attacks on encrypted images, a visual comparison is performed between the HAICDHBC algorithm and AES-CBC. As can be seen in Figure 5 and Figure 6, both exhibit damage caused by the same noise of the same size. However, in the case of AES-CBC, the decrypted image fails to provide meaningful information about the original image. Furthermore, the assessment of image sharpness using the SP under the influence of the four mentioned noises is detailed in Table 14. The image containing Sor Juana’s message is the most affected by noise. When the noise size is 50%, the sharpness value drops to 29%, while in other cases, it hovers around 55%. On the other hand, a 3 × 3 median filter was applied to the damaged images, and the results were reevaluated using the SP parameter. These results can be observed in Table 15. The most significant improvement was observed in Sor Juana’s message, with SP increasing from 29% to 57%, almost doubling in sharpness. Additionally, the Lena image exhibited an marked improvement, with the sharpness reaching up to 87%.

Another noteworthy aspect is the encryption quality. Table 16 presents a comparison of the entropy in this and other works for grayscale images. Notably, use of the HAICDHBC algorithm leads to an entropy very close to 8, surpassing other methods. However, it is essential to acknowledge that this improvement comes at a cost. The image size increases by approximately 8%, making the transmission of encrypted images difficult due to the larger size. Nevertheless, this trade-off results in an enhanced level of security.

Finally, it is highlighted that the values of the goodness-of-fit test in Table 11 are close to zero, and in some cases, they are precisely zero. These results indicate the random distribution of the encrypted information. This observation is consistent with the evaluations of correlation, energy, contrast, homogeneity, and the DFT shown in Table 4, Table 8, Table 9, Table 10 and Table 12, respectively. Therefore, the encryption is of a high quality.

## 8. Conclusions

In this paper, the hybrid cryptosystem HAICDHBC is introduced for image encryption, using ElGamal, the Diffie–Hellman protocol, the blockchain procedure with the Hash Sha-512 algorithm, and the number pi. The symmetrical system comprises fourteen rounds, incorporating dynamic substitution boxes, round keys, and permutations. An algorithm is also included to improve the entropy. The results demonstrate high-quality image encryption, evidenced by notably excellent results in entropy and goodness-of-fit tests. Comparative analyses with other works reveal a significant improvement in the entropy results. The algorithm’s resilience to noise attacks was assessed by damaging encrypted images with four types of noise at various intensities, and it demonstrated a superior resistance compared to AES–CBC. A novel parameter, SP, was introduced to evaluate damage and assess sharpness improvements with the application of a median 3 × 3 filter to damaged images. A security analysis affirms the algorithm’s resistance to known attacks, establishing its security. Future work will focus on developing a digital signature algorithm for images utilizing the number pi and the Diffie–Hellman protocol [64].

## Figures and Tables

**Table 1:** Probability estimation $P(x_{i})$ of a bit $x_{i}$ with different samples.

Chain Length	Percent of Zeros (%)	Percent of Ones (%)
$2^{3}$	75.000000	25.000000
$2^{10}$	51.074219	48.925781
$2^{15}$	49.935913	50.064087
$2^{20}$	50.023270	49.976730
$2^{25}$	49.990329	50.009671
$2^{30}$	49.999331	50.000669
$2^{35}$	50.000190	49.999810
$2^{40}$	50.000034	49.999966

**Table 2:** Values employed in this work.

Variable	Hexadecimal Value
$q_{1}$	C74A52C90C7095EC92B727D85CE31218 C3863BF9000DFDA1C3E0A284F3E7A700 4E793365586ACFAA79DC99FB627BF8CD 1E49A56863EBDAAD5701E025363D607
$q_{2}$	B0C1B9F894F6AC59082D91B8697E0689 6CD0C921161B445703B67B0F1AD3C5B1 858DDB6903723FB20FFA6608D8B3E656 AA003767762E010D1C769C876FB603A9
*k*	FF0
$p^{*}$	89104ED0230E59E3F4BB9575AFD05227B51EA7 EA635698ED6CFFE8A67E1BF72D96E128354BD A521E302B128C29B4E41B7381CDA8EC89E0BD AC049FFD8EA7865A7E5697E496EBC4DDCBE1 28ECD23A817BDCDE53684B479ACF1FCFABC C0416496FF978E82610BA253B11483D612D032E E24F44D6C3E1D70944E2F3CECD77C3AFEA411
α	2B62FB6FDE4EF4204BA91E06AA5B4E076BCC9 4A382C5B926F5DBC89F5E432DBC34A5565E15 D88E8956CF414B3DBBECC9DA928E3F92BD99 9DA7864B87ED884A5B309635DA0D6F00503B2 69192BE1FB84C504A067228E65B67E1C2C491 43C68F179BDC50DDCB4E7C378C43C0482501 FE6AFE00C8A91320D2963639A09D335796DD
$\beta_{A}$	9FECC2EC3057B87D5902733EDDC02F9A0687 525015F0EDFA99BABB65DFC8BBECB8E2B150 0767A267048E5CA01EB0EC87C14825BCBB3C2 01A67CFB616580308B09D5EF8FDBFF25397CA 0013BCBB3959DEEC18710531B26DAB9DE7468 DBD04DB76A213D8C39E8B18346B130D2A28C 44A2BB31A8C4CE7CE7A75E51A06F2A2F45239
$\beta_{B}$	37550E7CBF9338DAE5484461E73B56DC95F21 F4D43E9B3120B04C6F6450B345E73A63F597B3 922CD2D1F271B6B4773F6EB684FE938D8EF8E 6F3F39A7CE95D2DBAFCB104F1A1F2779B1F6 F34B5331AC7BD6B61902AED70C6C475AB79A 0412A36D13ADB900A6A7299B7B31D176E070F 670E7804754D5114459AAED3BF6765C5E5F426

**Table 3:** Entropy results per color of encrypted images.

Image	Red	Green	Blue
Sor Juana	7.9999999	7.9999981	7.9999982
Barbara	7.9999997	7.9999946	8.0
Lena	7.9999997	7.9999990	7.9999970
Baboon	7.9999998	7.9999991	7.9999996
Peppers	7.9999999	8.0	8.0
Donkey	8.0	7.9999997	7.9999997

**Table 4:** Correlation coefficient per color and direction of encrypted images.

Direction	Image	Red	Green	Blue
Horizontal	Sor Juana	0.00205	0.00700	0.00169
	Barbara	0.00219	−0.00311	0.00600
	Lena	−0.00768	0.00460	0.00370
	Baboon	−0.00216	−0.00419	−0.00804
	Peppers	0.00286	0.00025	0.00214
	Donkey	0.00102	0.00481	−0.00031
Vertical	Sor Juana	−0.00072	0.00126	0.00548
	Barbara	−0.00177	−0.00149	0.00275
	Lena	−0.00640	−0.00325	−0.00220
	Baboon	0.00046	−0.00011	−0.00131
	Peppers	0.00131	−0.00308	−0.00054
	Donkey	0.00267	0.00418	−0.00568
Diagonal	Sor Juana	−0.00549	−0.00340	−0.00191
	Barbara	0.00058	−0.00321	−0.00909
	Lena	0.00590	0.00520	0.00388
	Baboon	−0.00366	0.00543	0.00136
	Peppers	0.00655	−0.00379	−0.00315
	Donkey	0.00158	0.00658	−0.00678

**Table 5:** Number of Pixels Change Rate (NPCR) results per color of encrypted images.

Image	Red	Green	Blue
Sor Juana	99.63	99.59	99.61
Barbara	99.62	99.62	99.60
Lena	99.59	99.62	99.60
Baboon	99.60	99.61	99.61
Peppers	99.60	99.62	99.58
Donkey	99.61	99.63	99.62

**Table 6:** Unified Average Changing Intensity (UACI) results per color of encrypted images.

Image	Red	Green	Blue
Sor Juana	33.47	33.38	33.46
Barbara	33.44	33.54	33.47
Lena	33.49	33.48	33.49
Baboon	33.38	33.52	33.46
Peppers	33.44	33.52	33.48
Donkey	33.34	33.50	33.51

**Table 7:** Avalanche Criteria (AC) results per color of encrypted images.

Image	Red	Green	Blue
Sor Juana	50.03	49.97	49.98
Barbara	50.00	49.98	49.96
Lena	49.96	49.99	50.01
Baboon	49.95	50.00	50.02
Peppers	49.97	50.00	49.97
Donkey	49.96	50.00	50.01

**Table 8:** Energy results per color of encrypted images.

Image	Red	Green	Blue
Sor Juana	0.01563	0.01563	0.01563
Barbara	0.01563	0.01563	0.01563
Lena	0.01563	0.01563	0.01563
Baboon	0.01563	0.01563	0.01563
Peppers	0.01563	0.01563	0.01563
Donkey	0.01563	0.01563	0.01563

**Table 9:** Contrast results per color of encrypted images.

Image	Red	Green	Blue
Sor Juana	10.43	10.47	10.48
Barbara	10.49	10.52	10.53
Lena	10.47	10.50	10.53
Baboon	10.53	10.46	10.49
Peppers	10.50	10.51	10.49
Donkey	10.50	10.51	10.49

**Table 10:** Homogeneity results per color of encrypted images.

Image	Red	Green	Blue
Sor Juana	0.390	0.391	0.390
Barbara	0.389	0.389	0.389
Lena	0.389	0.389	0.388
Baboon	0.388	0.389	0.389
Peppers	0.389	0.389	0.389
Donkey	0.389	0.389	0.388

**Table 11:** Goodness-of-fit test (✓ Accept) with a rejection threshold of 308.

Image	Red	Green	Blue
Sor Juana	0.3/✓	1.2/✓	0.1/✓
Barbara	0.3/✓	0.2/✓	0.9/✓
Lena	1.6/✓	0.0/✓	0.0/✓
Baboon	0.0/✓	0.5/✓	0.1/✓
Peppers	0.0/✓	0.0/✓	0.2/✓
Donkey	0.0/✓	0.0/✓	0.0/✓

**Table 12:** Discrete Fourier Transform (DFT) evaluation (✓ Accept) with α=0.01.

Image	Red	Green	Blue
Sor Juana	0.470/✓	0.287/✓	0.392/✓
Barbara	0.148/✓	0.933/✓	0.571/✓
Lena	0.306/✓	0.423/✓	0.465/✓
Baboon	0.284/✓	0.815/✓	0.704/✓
Peppers	0.945/✓	0.988/✓	0.418/✓
Donkey	0.153/✓	0.883/✓	0.331/✓

**Table 13:** Entropy and correlation values for encrypted, completely black and white images.

Parameter	Image	Red	Green	Blue
Entropy	Black	7.9999993	7.9999997	7.9999994
	White	7.9999995	8.0	7.9999992
Horizontal Correlation	Black	0.00279	−0.00160	0.00703
	White	0.00663	−0.00243	0.00041
Vertical Correlation	Black	−0.00357	0.00699	−0.00295
	White	0.00069	0.00361	0.00032
Diagonal Correlation	Black	−0.00242	0.00087	−0.00511
	White	0.00583	0.00066	0.00644

**Table 14:** Similarity Parameter (SP) results for different size of $\chi^{2}$ noise applied.

Color	Image	20%	30%	40%	50%
Red	Sor Juana	72.69	58.16	43.53	29.27
	Barbara	82.70	73.86	64.14	55.96
	Lena	79.70	70.80	60.24	50.69
	Baboon	82.81	74.09	64.42	55.70
	Peppers	83.19	74.79	65.89	57.41
	Donkey	72.47	58.63	46.16	31.72
Green	Sor Juana	72.70	58.08	43.57	29.30
	Barbara	82.81	73.82	64.14	55.80
	Lena	81.16	72.86	63.24	54.37
	Baboon	83.56	75.26	66.10	57.84
	Peppers	80.26	70.29	59.69	49.71
	Donkey	72.13	58.16	45.83	31.00
Blue	Sor Juana	72.54	58.16	43.53	28.97
	Barbara	82.72	73.83	64.17	55.83
	Lena	83.07	75.58	66.78	58.57
	Baboon	82.14	73.13	63.07	54.09
	Peppers	79.77	69.51	58.74	48.86
	Donkey	72.58	58.64	46.04	31.84

**Table 15:** SP results after application of a 3 × 3 median filter after noise attacks of 50%.

Color	Image	Occlusion	Additive	Multiplicative	Chi-Square
Red	Sor Juana	58.65	57.62	58.26	57.10
	Barbara	81.12	81.43	81.66	81.24
	Lena	82.63	82.35	83.52	82.91
	Baboon	76.45	76.61	76.80	76.33
	Peppers	88.39	88.28	88.69	88.26
	Donkey	61.76	61.53	63.16	60.85
Green	Sor Juana	58.32	57.52	58.08	57.29
	Barbara	81.17	81.48	81.71	81.28
	Lena	84.24	84.20	84.99	84.39
	Baboon	76.90	76.99	77.23	76.81
	Peppers	81.72	81.61	82.28	81.53
	Donkey	61.09	61.00	62.66	59.85
Blue	Sor Juana	58.45	57.44	58.19	57.10
	Barbara	81.18	81.48	81.63	81.39
	Lena	87.76	87.82	88.30	87.90
	Baboon	73.99	74.21	74.43	73.96
	Peppers	80.98	80.57	81.47	80.76
	Donkey	61.72	61.51	63.23	60.64

**Table 16:** Entropy comparison with other works.

Image	Algorithm	Entropy
Lena	HAICDHBC	7.9999
	Ref. [59]	7.9992
	Ref. [60]	7.9993
	Ref. [61]	7.9994
Baboon	HAICDHBC	7.999999
	Ref. [62]	7.999800
	Ref. [17]	7.999800
	Ref. [63]	7.999900

## Data Availability

The data that support the findings of this study are available from the corresponding author upon request.

## Abbreviations

The following abbreviations are used in this manuscript:

AC	Avalanche Criteria
AES	Advanced Encryption Standard
DFT	Discrete Fourier Transform
HAICDHBC	Hybrid Information Encryption Algorithm using the Diffie–Hellman Protocol and Blockchain
NPCR	Number of Pixels Change Rate
SP	Similarity Parameter
UACI	Unified Average Changing Intensity
